# Chemokine Ligand 5 (CCL5) Derived from Endothelial Colony-Forming Cells (ECFCs) Mediates Recruitment of Smooth Muscle Progenitor Cells (SPCs) toward Critical Vascular Locations in Moyamoya Disease

**DOI:** 10.1371/journal.pone.0169714

**Published:** 2017-01-10

**Authors:** Ji Hoon Phi, Naoko Suzuki, Youn Joo Moon, Ae Kyung Park, Kyu-Chang Wang, Ji Yeoun Lee, Seung-Ah Choi, Sangjoon Chong, Reizo Shirane, Seung-Ki Kim

**Affiliations:** 1 Division of Pediatric Neurosurgery, Seoul National University Children’s Hospital, Seoul National University College of Medicine, Seoul, Republic of Korea; 2 College of Pharmacy, Sunchon National University, Sunchon, Republic of Korea; 3 Department of Anatomy, Seoul National University College of Medicine, Seoul, Republic of Korea; 4 Department of Neurosurgery, Miyagi Children’s Hospital, Sendai, Japan; European Institute of Oncology, ITALY

## Abstract

The etiology and pathogenesis of moyamoya disease (MMD) are still obscure. Previous studies indicated that angiogenic chemokines may play an important role in the pathogenesis of the disease. Recently, it was discovered that peripheral blood-derived endothelial colony-forming cells (ECFCs) and smooth muscle progenitor cells (SPCs) have defective functions in MMD patients. Therefore, the interaction of ECFCs and SPCs, the precursors of two crucial cellular components of vascular walls, with some paracrine molecules is an intriguing subject. In this study, co-culture of ECFCs and SPCs from MMD patients and healthy normal subjects revealed that MMD ECFCs, not SPCs, are responsible for the defective functions of both ECFCs and SPCs. Enhanced migration of SPCs toward MMD ECFCs supported the role for some chemokines secreted by MMD ECFCs. Expression arrays of MMD and normal ECFCs suggested that several candidate cytokines differentially produced by MMD ECFCs. We selected chemokine (C-X-C motif) ligand 6 (CXCR6), interleukin-8 (IL8), chemokine (C-C motif) ligand 2 (CCL2), and CCL5 for study, based on the relatively higher expression of these ligands in MMD ECFCs and their cognate receptors in MMD SPCs. Migration assays showed that only CCL5 significantly augmented the migration activities of SPCs toward ECFCs. Treatment with siRNA for the CCL5 receptor (CCR5) abrogated the effect, confirming that CCL5 is responsible for the interaction of MMD ECFCs and SPCs. These data indicate that ECFCs, not SPCs, are the major players in MMD pathogenesis and that the chemokine CCL5 mediates the interactions. It can be hypothesized that in MMD patients, defective ECFCs direct aberrant SPC recruitment to critical vascular locations through the action of CCL5.

## Introduction

Moyamoya disease (MMD) is an idiopathic intracranial vasculopathy. MMD is a leading cause of pediatric stroke, especially in East Asian countries. MMD is characterized by progressive stenosis and occlusion of the bilateral distal internal carotid arteries and their major branches [1]. Histopathological studies noted distinctive features in the disease-affected arteries: fibrocellular thickening of the intima, irregular undulation of the internal elastic lamina, and attenuation of the media [2]. The thickened intimal layer was mainly composed of smooth muscle cells [3]. The pathogenesis of MMD is still elusive despite the recent discovery of disease-linked genetic variation at the RNF213 locus [4].

Since the 1990s, researchers have investigated the role of angiogenic cytokines in moyamoya pathology [5, 6]. Earlier studies revealed that basic fibroblast growth factor (bFGF) and its receptor were highly expressed in the vessels of MMD patients [1]. Elevated plasma levels of vascular endothelial growth factor (VEGF) and platelet-derived growth factor BB (PDGF-BB) were also reported, reinforcing the concept of cellular component recruitment in the pathology of moyamoya by humoral and/or paracrine factors [7]. The concept was further supported by the discovery of circulating endothelial progenitor cells (EPCs) in peripheral blood and their crucial functions in the repair of the vascular endothelium in many vascular diseases, such as ischemic heart attack and stroke [8, 9]. It has been proposed that circulating EPCs participate in the development of MMD in a study using human autopsy specimens [10]. Previously, we reported that fewer EPCs were derived from the blood of pediatric MMD patients and that their EPCs have functional deficits compared with EPCs from healthy volunteers [11]. The data indicated that an aberrant (or defective) repair mechanism mediated by EPCs recruited into the critical vascular point in the carotid arteries may underlie the pathogenesis of MMD. The definition of EPCs cultured in vitro from blood has been challenged because cells with different characteristics can be included in the EPC culture; therefore, we will use the term endothelial colony-forming cells (ECFCs) to denote the outgrowth cells from the adherent culture of peripheral blood.

As noted above, the cardinal feature of moyamoya pathology is intimal thickening with smooth muscle cell proliferation. Therefore, the pathogenetic mechanism should include the recruitment of smooth muscle cells into the intimal layer where cellular proliferation and vascular occlusion occur. Recently, we isolated smooth-muscle progenitor cells (SPCs) from the peripheral blood of MMD patients and found that patient-derived SPCs had a disturbed tube formation capacity in vitro: they made significantly fewer tubes but these tubes exhibited a more thickened morphology [12].

Based on the data and results from our group and other groups, we hypothesized that some chemokines derived from defective ECFCs in MMD patients may be deeply involved in recruiting circulating SPCs into the intimal layer of carotid arteries. This inappropriate recruitment of SPCs and their proliferation may result in the fibrocellular thickening and vascular occlusion. The crosstalk between ECFCs and SPCs, the cellular components in moyamoya pathogenesis, was further investigated.

## Materials and Methods

### Study subjects

The study was performed with ECFCs and SPCs cultured from the peripheral blood of healthy volunteers (normal controls) and MMD patients. All patients were diagnosed with MMD by brain magnetic resonance imaging and digital subtraction angiography. Patients with other medical conditions (moyamoya syndrome), such as neurofibromatosis or hyperthyroidism, were excluded. Blood (40 ml) was obtained with informed consent. All blood samples were processed within 2 hr of collection. For mRNA expression microarray experiments, cells derived from 7 MMD patients and 4 healthy normal subjects were used. For the in vitro experiments, cells derived from 12 MMD patients and 7 healthy normal subjects were used. The presence of RNF213 c.14576G>A variation was verified by gene sequencing and confirmed with PCR-based mutation analysis (S1 Table). This study was approved by the Institutional Review Board of the Seoul National University Hospital.

### Cell cultures

The procedures for buffy coat isolation and ECFC culture were performed as previously described [11]. Mononuclear cells were isolated by density gradient centrifugation and the cells were plated in each well of a collagen-coated 6-well plate (BD BioCoat plate; BD Biosciences, San Jose, CA) with endothelial cell growth medium (catalog number CC-3162; Clonetics, San Diego, CA). For differentiation into an SPC lineage, PDGF-BB (5 ng/mL; R&D Systems, Minneapolis, MN) was added to the culture medium at one week [12]. The cells were maintained at 37°C in a humidified atmosphere containing 5% CO2.

### Phenotypic characterization

Fluorescence-activated cell sorting (FACS) analysis was performed by staining with 10 μl/ each of phycoerythrin (PE) or Fluorescein isothiocyanate (FITC) conjugated CD34 antibody (BD Biosciences), KDR/Flk-1 (VEGF receptor-2) antibody (R&D System Inc), VE-cadherin antibody (Bender MedSystem GmbH, Vienna, Austria), CD31 antibody (BD Biosciences), α-smooth muscle actin (α-SMA) antibody (R&D System Inc), Platelet-derived growth factor receptors (PDGFR-α) antibody, PDGFR-β antibody and CD45 antibody (BD Biosciences) for 30 min at 4°C in a dark room. Data were analyzed using a FACScan^®^ flow cytometer and CellQuest^®^ software (Becton Dickinson, Franklin Lakes, NJ). The ECFCs and SPCs were seeded onto an 8-well chamber glass microscope slide, and then were fixed in 1% paraformaldehyde and permeabilized with 0.1% Triton X-100. The cells were incubated with CD34 (SantaCruz, Dallas, TX), Von Willebrand factor (vWF), CD31, α-SMA (Abcam, Cambridge, UK), CD45 (BD Biosciences) and Calponin (DAKO, Glostrup, Denmark) antibodies for overnight at 4°C, and incubated with fluorescence-conjugated secondary antibodies for 1 hr at room temperature.

### Capillary tube-formation assay

For evaluation of angiogenic activity, ECFCs and SPCs were labeled with green fluorescent dye and red fluorescent dye (Sigma, St. Louis, MO). ECFCs and SPCs (2 × 10^4^ cells/well) were seeded onto 48-well culture plates coated with 50 μl of Matrigel (BD Biosciences) and incubated for 18 hr. The capillary tube-formations were visualized by fluorescence and quantified by counting the number of tubes using an inverted fluorescence microscope (Olympus BX-UCB; Olympus, Melville, NY). The tube thickness was measured in five random microscopic fields per subject using Image J software. Assays were performed in triplicate. We tested four different combinations of ECFCs and SPCs derived from healthy normal subjects and MMD patients.

### RNA extraction, gene expression profiling, and data analysis

Total RNA was extracted using Trizol reagent (Invitrogen, Carlsbad, CA) and purified using RNeasy mini kits (Qiagen, Mississauga, Canada). The integrity and concentration of RNA were determined by a NanoDrop spectrophotometer (NanoDrop Technologies, Wilmington, DE) and Agilent Bioanalyzer (Agilent Technologies, Santa Clara, CA). According to the manufacturer’s protocols, Illumina Human HT-12 BeadChips (48k) were used to generate expression profiles.

The raw data were transformed with a log2 method and normalized with a robust spline normalization (rsn) method using the R/Bioconductor lumi package [13]. Only the probes with a detection p-value < 0.01 in more than one sample were considered as detected probes. Finally, probes for the genes located on chromosome X or Y were excluded to remove the effect of sampling bias from gender-unmatched MMD and normal samples. A total of 16,103 probes (12,051 Entrez Gene IDs) were used for the detection of genes between ECFCs derived from MMD patients and normal ECFCs based on a Bayesian moderated t-test [14]. For genes with multiple probes, the probe with the largest SD was selected as the representative. The Benjamini and Hochberg (BH) false discovery rate (FDR) P value was calculated for multiple testing correction [15]. KEGG enrichment analysis was performed using DAVID Bioinformatics Resources (https://david.ncifcrf.gov/).

### Real-time quantitative-PCR analysis

The levels of mRNA expression were confirmed by real-time quantitative PCR (RT-qPCR) using an ABI 7000 Sequence Detection System (Applied Biosystems, Foster City, CA). The reactions were processed according to the manufacturer’s protocol with SYBR^®^ green master mix or TaqMan^®^ universal PCR master mix. Pre-designed Assays-on-Demand TaqMan^®^ probes and primer pairs were obtained from Applied Biosystems [Assay ID Hs00605742_g1 for chemokine (C-X-C motif) ligand 6 (CXCL6), Hs00174103_m1 for IL8, and Hs99999905_m1 for GAPDH]. The designed primer sequences (Cosmo Genetech, Seoul, Korea) were as follows: chemokine (C-C motif) ligand 2 (CCL2), Forward- 5’-CCC CAG TCA CCT GCT GTT AT-3’, and Reverse- 5’-AGA TCT CCT TGG CCA CAA TG-3’; CCL5, Forward- 5’-ACC ACA CCC TGC TGC TTT GC-3’, and Reverse- 5’-CCG AAC CCA TTT CTT CTC TGG-3’; CXCR1, Forward- 5’-TTT GTT TGT CTT GGC TGC TG-3’, and Reverse- 5’-AGT GTA CGC AGG GTG AAT CC-3’; CXCR2, Forward- 5’-ACA TGG GCA ACA ATA CAG CA-3’, and Reverse- 5’-TGA GGA CGA CAG CAA AGA TG-3’; CCR2, Forward- 5’-AGT TCA GAA GGT ATC TCT CGG TC-3’, and Reverse- 5’-GGC GTG TTT GTT GAA GTC ACT-3’; CCR5, Forward- 5’-GGA CCA AGC TAT GCA GGT GAC-3’, and Reverse- 5’-TTG GCA ATG TGC TTT TGG AA-3’; CCR1, Forward- 5’-CTG GTT GGA AAC ATC CTG GT-3’, and Reverse- 5’-GGA AGC GTG AAC AGG AAG AG-3’; CCR3, Forward- 5’-GTG TTC ACT GTG GGC CTC TT-3’, and Reverse- 5’-GTG ACG AGG AAG AGC AGG TC-3’; GAPDH, Forward- 5’-CAA TGA CCC CTT CAT TGA-3’, and Reverse- 5’-GAC AAG CTT CCC GTT CT-3’. Relative gene expression levels were determined from the Ct values obtained and using the 2-ΔΔCt method.

### Migration assay

The migration assay was performed using a trans-well membrane (8 μm pore size; Corning Costar Corp., New York, NY) in 24-well plates. The SPCs were seeded onto the upper chamber, and media containing various concentrations of recombinant human cytokines (rhCXCL6, rhIL8 and rhCCL5) or ECFCs were placed in the lower chamber. The plates were incubated for 24 hr, and the migrated cells were fixed and stained. Cells that had migrated to the lower surfaces of the membranes were quantified under a microscope. The same studies were conducted for CXCL6 and IL8. All assays were conducted in triplicate.

### Cell viability assay

Cell viability was measured by EZ-Cytox Cell Viability Assay Kit (DaeilLab Service, Seoul, Korea). The cells were seeded on 96-well plates (1 × 10^4^ cells/well) and were treated with the various concentrations of CXCL6, IL8 and CCL5. After incubation for 18 hr, cells were incubated with 10 μl of Ez-CyTox solution for 4 hr and measured using a microplate reader at 450 nm. The percentage of cell viability was presented by relative to the untreated control group. The experiments were performed in quintuplicate.

### Knockdown of CCR5 with siRNA

For siRNA transfection, normal and MMD SPCs were transfected with two CCR5 siRNAs (Ambion #4390843, Thermo Fisher Scientific, Waltham, MA; Bioneer #1172495, Bioneer, Daejeon, Korea) and two nonspecific control siRNAs (Ambion #AM16708 and Bioneer #SN-1002) using the RNAiMAX transfection reagent (Invitrogen).

### CCL5 Enzyme-linked immunosorbent assay (ELISA)

The concentrations of CCL5 in the plasma were measured by ELISA using commercially available kits (R&D Systems). Control and patient plasmas were harvested and centrifuged with a 10000xg for 10 min. Captured concentrations were read using an ELISA plate reader instrument. Assays were performed in duplicate for each sample.

### Statistical analysis

All data are expressed as the mean ± SD. All *P* values were two-sided and significance was set at p = 0.05. MedCalc version 12.4.0 (MedCalc, Ostend, Belgium; a free-trial version) was used for Kruskal–Wallis tests and IBM SPSS version 21.0 software (IBM, Armonk, NY) was used for t-tests.

## Results

### Characterization of ECFCs and SPCs

The peripheral blood mononuclear cells cultured on coated plates developed clusters of spindle-shaped cells after 7 days. These cells were maintained in two distinct differentiation conditions, one lacking PDGF-BB (for ECFC differentiation) and the other supplemented with PDGF-BB (for SPC differentiation). After 2–3 weeks, a cobblestone appearance and hill-and-valley morphology were observed by light microscopy as a sign of differentiation toward ECFCs and SPCs, respectively (S1A Fig).

For further characterization of the ECFCs and SPCs derived from peripheral blood, we performed FACS and fluorescence staining. FACS analyses of ECFCs and SPCs were obtained for CD34, KDR (hematopoietic stem cell marker), VE-cadherin, CD31 (endothelial cell marker), α-SMA, PDGFR-α, PDGFR-β (pericytes and smooth muscle cell marker) and CD45 (leukocyte marker). The ECFCs were CD34^weak^KDR^+^VE-cadherin^+^CD31^+^α-SMA^weak^PDGFR-α&β^weak^CD45^-^. The SPCs were CD34^weak^KDR^-^VE-cadherin^-^CD31^-^α-SMA^+^PDGFR-α&β ^+^CD45^-^ (S1B Fig). These markers showed similar positive frequencies in cells derived from healthy normal subjects and MMD patients (S2 Table). We also confirmed that ECFCs were positively stained for endothelial cell markers (vWF and CD31) and that SPCs expressed smooth-muscle cell markers (α-SMA and Calponin) (S1A Fig).

### Angiogenic capability of ECFCs and SPCs

**Fig 1:**
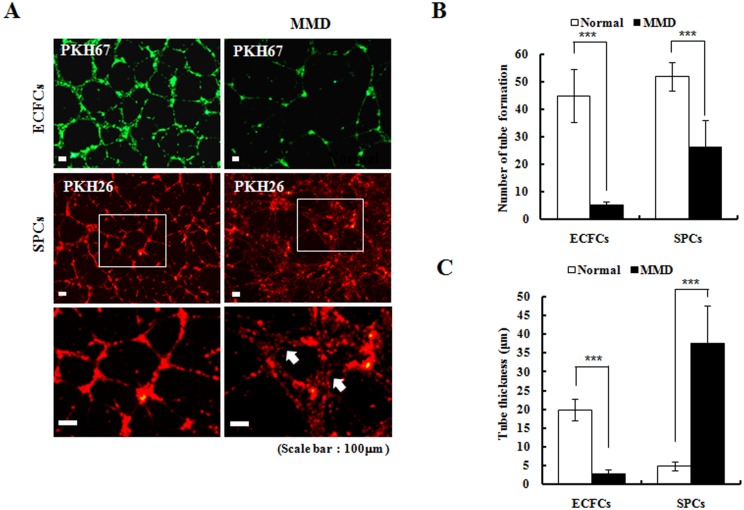
In vitro tube formation assays (original magnification ×100). (A and B) MMD ECFCs (green colored) make fewer tubes per unit area than normal ECFCs (n = 6, 11.9 ± 4.0% of control; p<0.001). MMD SPCs (red colored) also make less number of tubes (n = 6, 51.8 ± 21.5% of control; p<0.001). (C) The tube walls made by MMD ECFCs are thinner (n = 5, 14.4% of control; p< 0.001), but the tube walls composed of MMD SPCs are thicker than those of controls (n = 5, 763.4% of control; p<0.001) (*p<0.05, **p<0.01, ***p<0.001).

In vitro tube formation is an important functional capability of ECFCs and SPCs that reflect their angiogenic potentials. We compared the tube formation activity of these cells with that of normal controls and MMD patients. The tube structures made by the normal ECFCs were more organized than those made by the MMD ECFCs. The MMD ECFCs made fewer tubes per unit area than the normal ECFCs (Fig 1A and 1B). Moreover, the tube walls made by MMD ECFCs were thinner than those of the normal ECFCs (Fig 1C). The MMD SPCs also made fewer tube structures (Fig 1A and 1B), but the tube walls of the MMD SPCs were thicker than those made by the normal SPCs (Fig 1C).

### Essential role of normal ECFCs in angiogenesis

**Fig 2:**
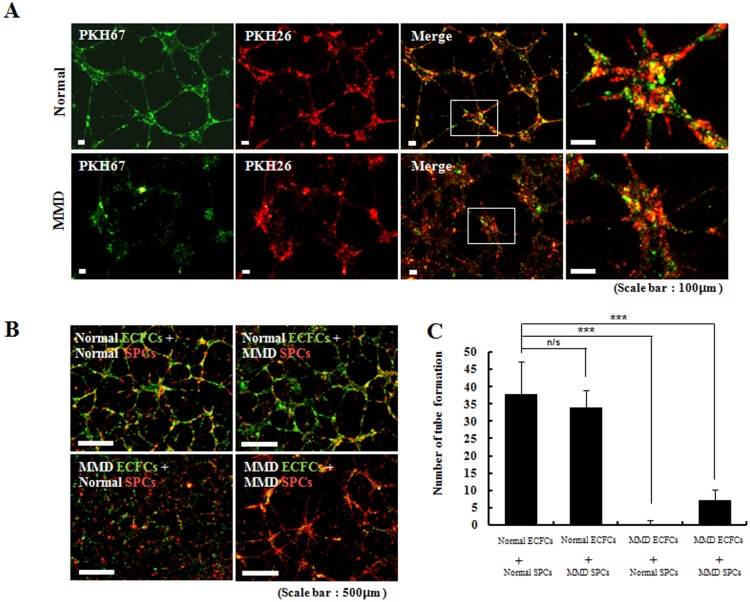
Co-culture experiments of ECFCs (green colored) and SPCs (red colored) (original magnification ×100). (A) In co-culture system (n = 7 for each group), both ECFCs and SPCs contribute to the tube formation. (B and C) MMD ECFCs when co-cultured with either normal SPCs or MMD SPCs make significantly less number of tubes than normal ECFCs (p = 0.168 for [normal ECFCs + normal SPCs] vs. [normal ECFCs + MMD SPCs]; p<0.001 for [normal ECFC+ normal SPC] vs. [MMD ECFC+ normal SPC]; p<0.001 for [normal ECFC+ normal SPC] vs. [MMD ECFC+ MMD SPC]).

When the ECFCs and SPCs were co-cultured on plate at an optimal cell density, we observed that both cell types contributed to the formation of tubes. In a double-staining immunofluorescence experiment, the normal ECFCs and normal SPCs contributed equally to tube formation. However, the MMD ECFCs and MMD SPCs made thicker tube structures than the normal cells, and these tube structures were composed mainly of MMD SPCs (Fig 2A).

We further measured the efficacy of in vitro tube formation under several co-culture conditions as follows: [normal ECFCs + normal SPCs], [normal ECFCs + MMD SPCs], [MMD ECFCs + normal SPCs], and [MMD ECFCs + MMD SPCs]. The co-cultures of [normal ECFCs + normal SPCs] and [normal ECFCs + MMD SPCs] formed intact tube networks, and the tubes formed consisted of the two types of cells. In contrast, the [MMD ECFCs + MMD SPCs] formed far fewer tubes than the [normal ECFCs + normal SPCs]. The tubes made by [MMD ECFCs + MMD SPCs] mainly originated from the MMD SPCs. Moreover, the co-culture of [MMD ECFCs + normal SPCs] failed to generate tube networks and only a minimal number of tube structures was observed (Fig 2B). The observed number of tubes per unit area was significantly different among groups (Fig 2C). Collectively, these observations indicated that the interaction of ECFCs and SPCs is an important factor in the formation of tube networks and suggested that normal-functioning ECFCs may be the essential contributor to angiogenesis.

### MMD ECFCs promote migration of SPCs

**Fig 3:**
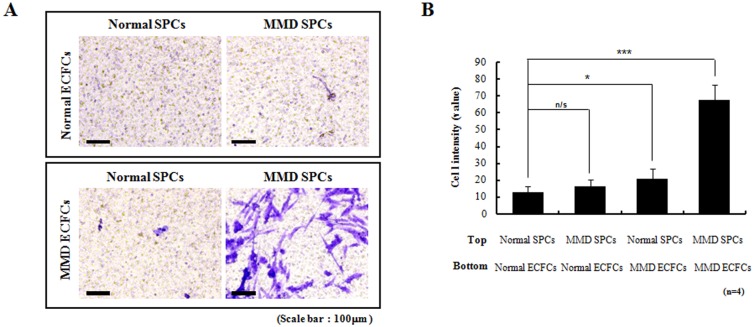
Trans-well migration assays (original magnification ×200). Migration of SPCs is enhanced when MMD ECFCs are placed in the bottom well (n = 4 for each group). The cell intensity value for combination of normal SPCs and MMD ECFCs is 166 ± 58.9% of control (combination of normal SPCs and normal ECFCs) (p = 0.029). The cell intensity for combination of MMD SPCs and MMD ECFCs are 539 ± 161.8% of control (p<0.001).

Because the co-culture of [MMD ECFCs + normal SPCs] failed to generate tube networks despite the intact capability of tube-formation by normal SPCs alone, we reasoned that MMD ECFCs may exert some effects on SPCs by humoral/paracrine factors. In a trans-well migration assay, no difference in migration was found between the normal SPCs and the MMD SPCs if the normal ECFCs were placed in the bottom wells. However, migration increased if the MMD ECFCs were situated in the bottom wells, indicating the secretion of some humoral factors by the MMD ECFCs. The augmentation of migration was highlighted when the MMD SPCs were combined with the MMD ECFCs (Fig 3).

### Profiling of the expression of chemokines in ECFCs from normal controls and MMD patients

**Fig 4:**
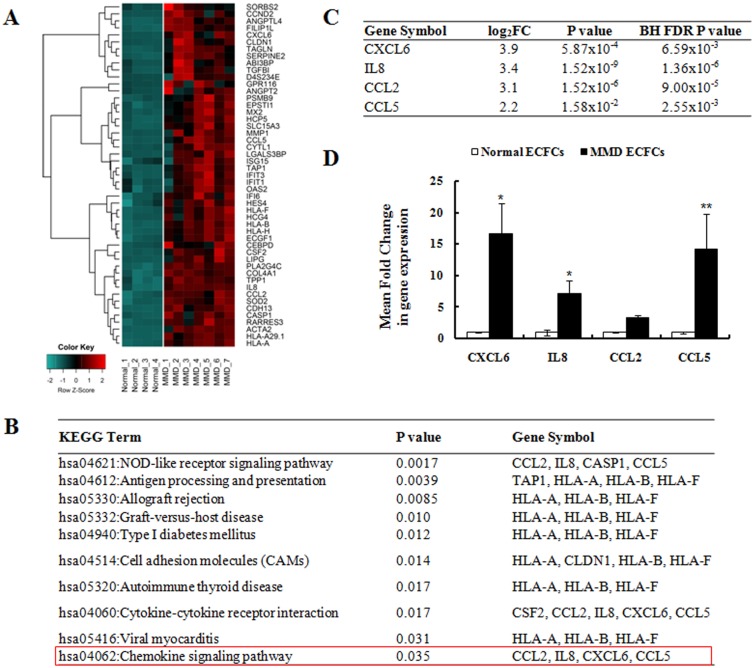
Differentially expressed chemokines in MMD ECFCs. (A) Expression of various chemokines are compared between normal ECFCs (n = 4) and MMD ECFCs (n = 7). (B) KEGG pathway enrichment analysis; upregulated genes are involved in chemokine signaling pathway. (C) Four most-up-regulated chemokines in MMD ECFCs are selected for further analysis. (D) Confirmation of mRNA expression of select gene in ECFCs by RTq-PCR (n = 4 for each group). Significantly higher levels of CLCL6, IL8, and CCL5 mRNA are expressed in MMD-ECFCs (CXCL6, p = 0.038; IL8, p = 0.021; CCL2 p = 0.239; CCL5, p = 0.008).

Tube formation is a multi-stage process involving the cellular adhesion, migration, and differentiation of contributing cells. We hypothesized that some humoral factors mediate the interaction of ECFCs and SPCs during angiogenic activity. To elucidate the interaction mechanism of the two cell types, we examined mRNA expression profiles and focused on 49 genes that were highly upregulated (fold change ≥4, BH FDR P value < 0.05) in the ECFCs from MMD patients compared with those from normal controls (Fig 4A). KEGG enrichment analysis revealed that the upregulated genes were enriched with genes involved in the chemokine signaling pathway (Fig 4B), including four differentially expressed chemokines (Fig 4C). The mRNA expression levels of selected genes were further confirmed by RTq-PCR. CXCL6, IL8, and CCL5 mRNA expression was significantly increased in the MMD ECFCs compared with the normal ECFCs (Fig 4D).

Then, we measured the mRNA expression of six chemokine receptors in MMD SPCs that interact with the selected four chemokines (S2 Fig). In the MMD SPCs, the mRNA expression of the receptors for CXCL6 (CXCR1, CXCR2), IL8 (CXCR1, CXCR2) and CCL5 (CCR5) was significantly increased by more than 2-fold. Therefore, we selected CXCL6, IL8 and CCL5 as candidate molecules medicating the interaction.

### CCL5 promotes SPC migration in MMD patients

**Fig 5:**
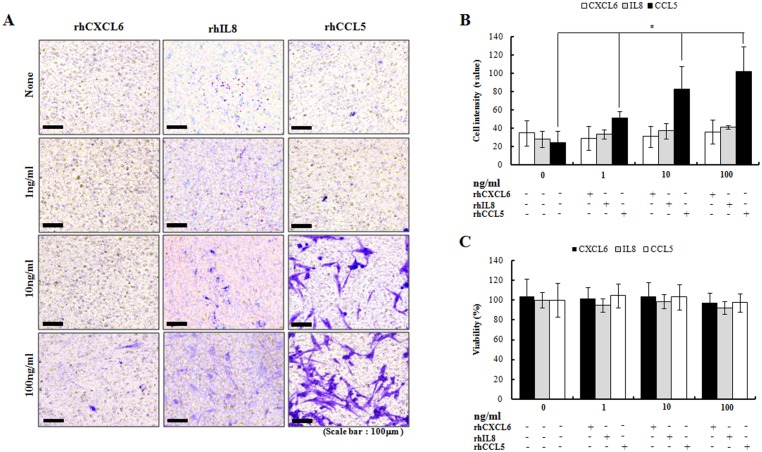
Augmentation of SPCs migration by rhCCL5 (original magnification ×200). (A and B) Addition of rhCCL5, not of CXCL6 or IL8 enhances the migration of MMDSPCs across the trans-well membranes without ECFCs in the bottom wells (n = 3 for each group). A dose-dependent increase of migration is observed (p = 0.027). (C) There is no difference in cell viability with the addition of rhCXCL6, rhIL8 or rhCCL5 (n = 5 for each group).

The relatively high expression of CXCL6, IL8 and CCL5 in MMD ECFCs and their cognate receptors in the MMD SPCs, prompted us to explore their roles. We examined whether recombinant CXCL6, IL8 or CCL5 could promote SPC migration in the trans-well system. The normal SPCs and MMD SPCs were incubated with various concentrations of recombinant human CXCL6 (rhCXCL6), IL8 (rhIL8), and CCL5 (rhCCL5), and the trans-membrane migration activity was measured. rhCXCL6 and rhIL8 induced a few normal SPCs and MMD SPCs to migrate toward SPCs, but rhCCL5 induced a robust increase in the migration of MMD SPCs (Fig 5A). Importantly, the increase in migration occurred in a dose-dependent manner (Fig 5B). In contrast, rhCCL5 did not induce migration of normal SPCs (S3 Fig). There was no difference in cell viability after the addition of rhCXCL6, rhIL8 or rhCCL5 (Fig 5C). Interestingly, plasma CCL5 levels were higher in MMD patients than control group (S4 Fig).

### Essential involvement of the CCL5-CCR5 interaction in the migration of SPCs from MMD patients

**Fig 6:**
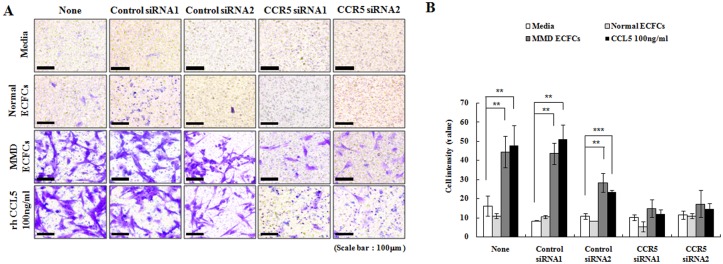
Blocking CCL5-CCR5 interaction with siRNA (original magnification ×200). (A) In trans-well migration assays (n = 3 for each group), treatment of CCR5 siRNAs effectively abrogates the enhanced migration of MMD SPCs by the presence of MMD ECFCs or addition of rhCCL5. (B) Quantification of migrated cells.

Our results suggested that the CCL5-CCR5 interaction was a crucial factor in SPC migration toward ECFCs in MMD patients. Therefore, we confirmed the validity of this mechanism by the knockdown of CCR5 in SPCs using RNA interference. To determine the efficiency of CCR5 siRNA, we measured expression levels of CCR5 by RTq-PCR (S5A Fig). In trans-well migration assays, CCR5 siRNA effectively inhibited the significantly enhanced migration of MMD SPCs by the presence of MMD ECFCs or rhCCL5 (Fig 6A and 6B). Next, we examined whether the knockdown of CCR5 in SPCs influenced their angiogenic activity in co-culture systems. The tube formation capability of SPCs after siRNA treatment was no different than that of the SPCs untreated with siRNA, indicating that the angiogenic activity of SPCs is not affected by inhibition of CCR5 (S5B Fig). These observations suggested that the CCL5-CCR5 interactions contribute to the migration of SPCs toward EPCs in MMD, which would eventually result in neointimal hyperplasia.

## Discussion

The causative factor or pathogenetic mechanism of MMD is one of the Holy Grails for researchers engaged in cerebrovascular diseases. Although various genetic and environmental factors have been proposed as the cause, MMD is still defined as ‘idiopathic’ occlusion of intracranial arteries [6]. Overt familial tendency and ethnic preference indicate the presence of genetic predisposition. Discovery of a susceptibility gene, RNF213, linked to familial and sporadic MMD in East Asian populations strongly corroborates a genetic predisposition to the disease [16]. Thus, it can be assumed that a pathologic vascular occlusion is triggered by some environmental factor in the setting of MMD susceptibility.

For a long time, researchers have focused on the angiogenic factors and pro-inflammatory molecules in the vascular walls, blood, and cerebrospinal fluid (CSF) of MMD patients. bFGF was the first molecule of this type to be investigated in vessel specimens of MMD patients [5]. Elevation of bFGF expression was noted in the walls of carotid arteries in autopsied patients and in the superficial temporal arteries in operative specimens [1]. bFGF is a potent angiogenic factor that can promote abnormal vessel formation in MMD. It is also noteworthy that bFGF can induce chemotaxis in smooth muscle cells [17]. We previously reported that matrix metalloproteinase (MMP)-9, monocyte chemotactic protein 1 (MCP1, also known as CCL2), interleukin-1β, and VEGF were elevated in the serum of MMD patients [7]. A polymorphism in the promoter of the tissue inhibitor of metalloproteinase (TIMP)-2 gene locus has been observed in familial MMD patients [18]. TIMP-2 impedes the activities of various types of MMPs including MMP-9. It is also intriguing that cellular retinoic acid binding protein (CRABP), a potent regulator of cytokines, is elevated in the CSF of MMD patients [19]. Therefore, aberrant activation or inhibition of some types of angiogenic/ chemotactic factors can be deeply involved in moyamoya pathogenesis. However, global elevation of some molecules in the serum and/or CSF cannot explain the focal and location-specific nature of MMD pathophysiology. It is still unclear whether elevation of angiogenic cytokine levels is a causative factor or just an epiphenomenon accompanying brain ischemia and stroke [20].

The key histological feature of moyamoya-involved vessels found in autopsy specimens is thickening of the intimal layers [2]. It is known that this thickened layer is mainly composed of smooth muscle cells [3]. However, further research into this field was hampered by the lack of appropriate animal models for MMD. The paucity of autopsy specimens of the disease-affected carotid vessels also added to the difficulty. Circulating progenitor cells, ECFCs, can participate in normal endothelial repair when a vascular injury occurs [21]. It is highly plausible that ECFCs play a major role in the occlusion of carotid arteries in MMD. There are conflicting results for the number of ECFCs found in the peripheral blood of MMD patients. We previously reported decreased numbers and defective angiogenic functions of ECFCs derived from pediatric MMD patients compared with controls [11, 22]. In contrast, Jung et al. [23] reported a higher frequency of ECFC outgrowth in MMD patients than in controls. Rafat et al. [24] also reported similar results. This controversy may reflect a different age distribution, heterogeneous disease stages of the study subjects, and differing culture conditions in each study. In fact, Jung et al. indicated that MMD patients had fewer colony-forming units than their controls and that significantly fewer outgrowth cells were derived from the late-stage MMD patients than the patients in earlier stages [23].

It has been suggested that smooth muscle cells in the neointima of atherosclerosis patients are derived from the bone marrow, through circulating SPCs [25]. Recently, we reported the successful isolation of circulating SPCs from MMD patients and found that MMD SPCs recapitulate the pathologic findings in the cerebral arteries of MMD patients [12]. We postulated that an interaction may exist between circulating ECFCs and SPCs during re-endothelialization and repair processes in the critical locations of MMD patients and that some chemokines can mediate the interaction. Endothelial CXCR4 is known to mediate the recruitment of circulating SPCs to vascular injury sites through CXCL12 and disruption of a CXCL12/CXCR4 axis leads to neointimal hyperplasia [26].

We attempted to identify the interaction patterns between ECFCs and SPCs from MMD patients and normal subjects. The disease-affected ECFCs and SPCs were less effective with respect to tube formation, as was expected from previous studies. We observed dual contributions from ECFCs and SPCs to making capillary tubes in vitro. Notably, the MMD ECFCs exerted more deleterious effects on the efficiency of tube formation than the MMD SPCs in combinational co-culture experiments. The [MMD ECFCs + normal SPCs] made far fewer tubes per unit area than the [normal ECFCs + MMD SPCs]. Furthermore, the MMD ECFCs enhanced the migration of MMD SPCs in the trans-well assay, but the normal ECFCs did not. We postulated that disease-affected ECFCs can secrete some humoral/paracrine factors to recruit SPCs to the lesion site. Expression arrays of MMD EPCs revealed 4 candidate chemokines involved in the chemokine signaling pathway, CXCL6, IL8, CCL2, and CCL5. We further narrowed the list of candidate molecules according to the relative expression of their receptors in MMD SPCs. Cognate receptors for CXCL6, IL8, and CCL5 showed 2-fold increased expression in MMD SPCs. Migration assays using rhCXCL6, rhIL8, and rhCCL5 revealed that only treatment with rhCCL5 produced robust enhancement of SPC migration toward ECFCs. Blocking the expression of the receptor for CCL5 (CCR5) with specific siRNAs abrogated the enhanced migratory effects.

CCL5 is also known to be involved in the progression of atherosclerotic plaques [27]. CCL5 belongs to the CC chemokine family which also includes CCL2. CCL2 was one of the candidate chemokines in our expression arrays of the MMD and normal ECFCs. CCL2 was also one of the cytokines elevated in the serum of MMD patients [7]. CCL5 has been associated with the progression of atherosclerosis, mediating smooth muscle cell activation and macrophage recruitment [27]. Polymorphisms in the CCL5 gene were associated with stroke risks [28, 29]. We postulated that impaired endothelial repair at the critical vascular location (distal internal carotid arteries) by disease-affected ECFCs and concomitant secretion of CCL5 lead to SPC recruitment to the lesion site and neointimal hyperplasia in MMD.

## Conclusions

We found that MMD-affected ECFCs rather than SPCs are critical to phenomenon of impaired tube formation in vitro. CXCL6, IL8, CCL2 and CCL5 are highly expressed in ECFCs derived from MMD patients. Through in vitro migration assays, we discovered that CCL5 is the main chemokine mediating SPC recruitment. Although both ECFCs and SPCs showed defective functions in previous studies, these data indicated that ECFCs, not SPCs are the major players in MMD pathogenesis. We propose that defective ECFCs in MMD patients direct the aberrant recruitment of SPCs to critical vascular locations through the action of CCL5.

## Supporting Information

S1 Fig Characterization of ECFCs and SPCs from the peripheral blood. (A) Cobble-stone (upper) and hill-and-valley (lower) appearance which are characteristic for ECSFs and SPCs, respectively. ECFCs are positive for endothelial cell markers (vWF and CD31). SPCs express smooth-muscle cell markers (α-SMA and Calponin, original magnification ×200). (B) FACS analyses show that ECFCs are CD34^weak^KDR^+^VE-cadherin^+^CD31^+^α-SMA^weak^PDGFR-α&β^weak^CD45^-^. SPCs are CD34^weak^KDR^-^VE-cadherin^-^CD31^-^α-SMA^+^PDGFR-α&β ^+^CD45^-^.
(EPS)

S2 Fig Comparison of mRNA expression of six chemokines receptors in MMD SPCs and normal SPCs (n = 3 for each group). CXCR1, CXCR2 (receptors for CXCL6 and IL8), and CCR5 (receptor for CCL5) show more than 2 folds increased expression in MMD SPCs.
(EPS)

S3 Fig The migration of normal SPCs by CCL5. Migration of normal SPCs is not increased after the treatment of 1, 10, and 100 ng/ml rhCCL5.
(EPS)

S4 Fig Secretory CCL5 levels are detected by ELISA assay. CCL5 levels are significantly higher in MMD plasma (n = 42) compared to normal control plasma (n = 19) (p = 0.001).
(EPS)

S5 Fig SPCs are transfected with siRNA specific for CCR5. (A) Effective knockdown of CCR5 by specific siRNAs are confirmed at the mRNA level in SPCs. (B) Tube formation assays in co-culture systems show decreased tube formation when MMD ECFCs and/or MMD SPCs are combined. CCR5 siRNA does not interfere tube formation (original magnification ×200).
(EPS)

S1 Table Sex and age of the moyamoya disease (MMD) patients and healthy normal subjects with information on the RNF213 c.14576 polymorphism. (DOCX)

S2 Table The percentages of marker-positive cells in ECFCs and SPCs. (DOCX)
