# Supplementary material for: Chemokine Ligand 5 (CCL5) Derived from Endothelial Colony-Forming Cells (ECFCs) Mediates Recruitment of Smooth Muscle Progenitor Cells (SPCs) toward Critical Vascular Locations in Moyamoya Disease
Source: PLoS One. 2017 Jan 10;12(1):e0169714. doi: 10.1371/journal.pone.0169714 (PMC5224827; doi:10.1371/journal.pone.0169714)
Supplement: S1 Table — (DOCX) [file pone.0169714.s006.docx]

S1 Table. Sex and age of the moyamoya disease (MMD) patients and healthy normal subjects.

|  | No | Sex | Age (yr) | Microarray data | in vitro experiments | RNF213  variant |
| --- | --- | --- | --- | --- | --- | --- |
| MMD patients | 1 | F | 7 | + | - | G/A |
|  | 2 | F | 8 | + | - | NA |
|  | 3 | F | 8 | + | - | G/A |
|  | 4 | F | 19 | + | - | G/A |
|  | 5 | F | 37 | + | - | G/A |
|  | 6 | M | 8 | + | + | NA |
|  | 7 | F | 36 | + | + | G/A |
|  | 8 | M | 1 | - | + | G/A |
|  | 9 | F | 4 | - | + | A/A |
|  | 10 | M | 7 | - | + | G/G |
|  | 11 | F | 17 | - | + | G/G |
|  | 12 | F | 19 | - | + | NA |
| Healthy normal subjects | 1 | M | 20 | + | + | G/A |
|  | 2 | F | 21 | + | + | G/G |
|  | 3 | F | 24 | + | + | G/G |
|  | 4 | M | 25 | + | + | G/G |
|  | 5 | F | 21 | - | + | G/G |
|  | 6 | M | 22 | - | + | G/G |
|  | 7 | F | 25 | - | + | G/G |

G/G: wild type (genotype GG),

G/A: heterozygote (genotype GA),

A/A: homozygote (genotype AA)

NA: Not Applicable
