# Supplementary material for: Chemokine Ligand 5 (CCL5) Derived from Endothelial Colony-Forming Cells (ECFCs) Mediates Recruitment of Smooth Muscle Progenitor Cells (SPCs) toward Critical Vascular Locations in Moyamoya Disease
Source: PLoS One. 2017 Jan 10;12(1):e0169714. doi: 10.1371/journal.pone.0169714 (PMC5224827; doi:10.1371/journal.pone.0169714)
Supplement: S2 Table — (DOCX) [file pone.0169714.s007.docx]

S2 Table. The percentages of marker-positive cells in ECFCs and SPCs.

|  | ECFCs | | SPCs | |
| --- | --- | --- | --- | --- |
|  | Normal | MMD | Normal | MMD |
| CD34 | 22.24± 23.05% | 13.22 ± 10.60% | 2.58 ± 1.73% | 7.18 ± 6.07% |
| KDR | 81.78± 3.20% | 76.87± 16.47% | 2.15 ± 0.30% | 8.32 ± 6.54% |
| VE-cadherin | 70.99± 25.50% | 69.12± 3.56% | 3.04 ± 1.45% | 4.07 ± 1.60% |
| CD31 | 99.73 ± 0.29% | 99.01 ± 0.62% | 4.25 ± 6.84% | 6.14± 10.61% |
| α-SMA | 26.14 ± 17.07% | 33.57 ± 20.32% | 86.39 ± 11.83% | 80.92 ± 29.99% |
| PDGFR-α | 0.98 ± 0.01% | 0.81 ± 0.81% | 53.65 ± 4.08% | 49.74 ± 18.68% |
| PDGFR-β | 11.20 ± 4.88% | 2.57 ± 1.65% | 81.12 ± 0.70% | 81.71 ± 1.20% |
| CD45 | 1.59 ± 1.42% | 1.23 ± 1.73% | 1.46 ± 0.81% | 1.89 ± 1.08% |

ECFCs: endothelial colony-forming cells

SPCs: smooth muscle progenitor cells

MMD: moyamoya disease

CD34: cluster of differentiation 34

KDR: kinase Insert domain receptor

VE-cadherin: vascular endothelial cadherin

CD31: cluster of differentiation 31

α-SMA: alpha smooth muscle actin

PDGFR-α: platelet-derived growth factor receptor alpha

PDGFR-β: platelet-derived growth factor receptor beta

CD45: cluster of differentiation 45 (Leucocyte Common Antigen)
